# Using Deep Learning for Classification of Lung Nodules on Computed Tomography Images

**DOI:** 10.1155/2017/8314740

**Published:** 2017-08-09

**Authors:** QingZeng Song, Lei Zhao, XingKe Luo, XueChen Dou

**Affiliations:** School of Computer Science & Software Engineering, Tianjin Polytechnics University, Tianjin, China

## Abstract

Lung cancer is the most common cancer that cannot be ignored and cause death with late health care. Currently, CT can be used to help doctors detect the lung cancer in the early stages. In many cases, the diagnosis of identifying the lung cancer depends on the experience of doctors, which may ignore some patients and cause some problems. Deep learning has been proved as a popular and powerful method in many medical imaging diagnosis areas. In this paper, three types of deep neural networks (e.g., CNN, DNN, and SAE) are designed for lung cancer calcification. Those networks are applied to the CT image classification task with some modification for the benign and malignant lung nodules. Those networks were evaluated on the LIDC-IDRI database. The experimental results show that the CNN network archived the best performance with an accuracy of 84.15%, sensitivity of 83.96%, and specificity of 84.32%, which has the best result among the three networks.

## 1. Introduction

Lung cancer which is the most common cancer in both men and women is a major burden of disease worldwide [1]. Some report estimated that the number of new cases of lung cancer is about 221,200, accounting for about 13% of all cancer diagnoses in 2015. The mortality of lung cancer accounts for about 27% of all cancer deaths [2]. For those reasons, lung nodules need to be examined and watched closely when it might be at an early stage. By the early detection, the 5-year survival rate of patients with lung cancer can be improved by about 50%.

Computed tomography (CT) is the most effective method of lung nodule detection for its ability to form three-dimensional (3D) images of the chest, resulting in greater resolution of nodules and tumor pathology. A CT image by computer processing to assist lung nodule diagnostics has been widely used in clinic. The process of computer-aided diagnosis (CAD) of lung cancer can be divided into a detection system (often abbreviated as CADe) and diagnostic system (often abbreviated as CADx). The CADe system divides the candidate nodules identified in the previous step into nodules or nonnodules (i.e., normal anatomic structures). The goal of the CADx system is to classify detected nodules into benign and malignant nodules [3]. Since the probability of malignancy is closely related to the geometric size, shape, and appearance, CADx can distinguish the benign and malignant pulmonary nodules by the effective features such as texture, shape, and growth rate. Thus, the success of a particular CADx system can be measured in terms of accuracy of diagnosis, speed, and automation level [4].

In recent years, neural networks, rebranded as “deep learning,” began beating traditional AI in every critical task: recognizing speech; characterizing images; and generating natural, readable sentences. Deep learning not only accelerates the critical task but also improves the precision of the computer and the performance of CT image detection and classification.

In this paper, the problem of classification of benign and malignant is considered. It is proposed to employ, respectively, the convolution neural network (CNN), deep neural network (DNN), and stacked autoencoder (SAE). The work can be used as input directly to reduce the complex reconstruction of data in the process of feature extraction and classification.

The rest of the paper is organized as follows.  Section 2 analyzes the related works.  Section 3 presents the proposed methodology for the classification of lung nodules. The experimental results obtained are discussed in Section 4. The conclusion of this paper was made in Section 5.

## 2. Related Works

Various initiatives are frequently developed aiming at increasing the accuracy of lung cancer diagnosis using a neural network. Chen et al. [5] proposed a method that uses a neural network ensemble (NNE) scheme to distinguish probably benign and uncertain and probably malignant lung nodules. Experimental results illustrated that the scheme had classification accuracy (78.7%) which is better than that of the individual classifier (LVQNN: 68.1%).

In [6], Kuruvilla and Gunavathi proposed a methodology based on texture features using the artificial neural network (ANN), with an accuracy rate of 93.30%. Using the combination of texture and shape features for detection and classification may result in improved classification accuracy [7]. Kumar et al. presented a methodology using the stacked autoencoder (SAE), a deep learning technique, with an accuracy rate of 75.01% [8].

Deep learning is based on using “deep” neural networks comprised of a large number of hidden layers. The deep belief network (DBN) which has undirected connections between its top two layers and downward-directed connections between all its lower layers [9] has been tested for classification of malignancy of lung nodules without computing the morphology and texture features [10]. It had reached the sensitivity rate of 73.40% and the specificity rate of 82.20% using the deep belief network.

Some research papers applied deep CNNs for detection or classifications of a medical image. In 2015, Shen et al. [11] diagnosed lung cancer on the LIDC database using a multiscale two-layer CNN and the reported accuracy was 86.84%. In [12], Shin et al. exploit and extensively evaluate three important, previously understudied factors on CNN architecture, dataset characteristics, and transfer learning.

## 3. Materials and Methods

In this section, the proposed approach on the LIDC-IDRI [13] dataset from the Lung Image Database Consortium is evaluated. The complex steps of image feature extraction in traditional medicine can be reduced by directly inputting the original image.

### 3.1. Convolution Neural Networks (CNNs)

A convolution neural network (CNN) is a multilayer neural network, which comprised of one or more convolution layers and then followed by one or more fully connected layers as in a standard multilayer neural network. The CNN was proposed in 1960s, with the ideas like local perception, the weights of sharing, and sampling in space or time. Local perception can find some local characteristics of the data for the basic features of the visual animals, such as an angle and an arc in the picture [14]. It is a kind of an efficient identification method which has attracted wide attention recently. The benefit of CNNs is that they are easier to train and have many fewer parameters than fully connected networks with the same number of hidden units.

Convolution neural network architecture is usually used in collaboration with the convolution layer and pool layer [15]. The affection of the pooling layer is to confuse the features of the specific position. Since some location features are not important, it just needs other features and the relative position. The pooling layer operation consists of max pooling and mean pooling. Mean pooling calculates the average neighborhood within the feature points, and max pooling calculates the neighborhood within a maximum of feature points. The error of feature extraction mainly comes from two aspects: the neighborhood size limitation caused by the estimated variance and convolution layer parameter estimated error caused by the mean deviation. Mean pooling can reduce the first error, retaining more image background information. Max pooling can reduce the second error, retaining more texture information.

**Figure 1:**
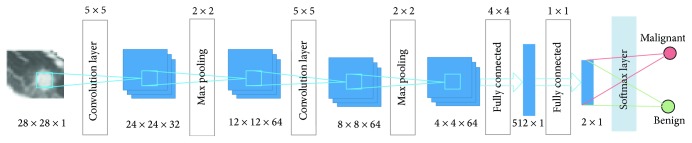
The architecture of the CNN.

The architecture of the CNN in this paper is showed in Figure 1. It is composed of multiple maps in each layer; each map is composed of multiple neural units, all the neural units in the same map share one convolution kernel (i.e., weight), and each convolution kernel represents a feature, such as access to the edge of image features. The detail of the CNN is showed in Table 1. The input data (image data) has a strong robustness on the distortion. The multiscale convolution image feature is generated by setting the convolution kernel size and parameter; the information of different angles is generated in the feature space.

### 3.2. Deep Neural Network (DNN)

A DNN is an increase in the number of hidden nodes in a simple neural network. The neural network can be used to carry on the more complex input calculation, because each hidden layer can be the nonlinear transformation of the output layer and the deep neural network is better than the “shallow” network. The nonlinear *f*(*x*) should be used for each hidden layer, because if the activation function is linear, compared with the single hidden layer neural network, the depth of the hidden layer of the network does not enhance the ability to express. The processing part of the pulmonary nodule is decomposed into the DNN, so that different network layers can be used to obtain the characteristics of the pulmonary nodules with different sizes. There are also local extremum problems and gradient diffusion problems in the DNN.

**Figure 2:**
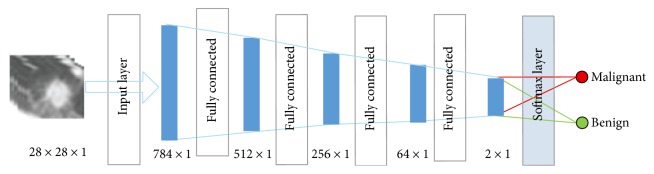
The architecture of the DNN.

In the training process, the original image is used as the input layer parameters, so as to retain a large amount of detailed information of the image. The input layer, hidden layer, and output layer of the DNN architecture are all connected layers, and the DNN does not contain a convolution layer. DNN training images and label was input into the DNN architecture; each layer of the weight in the first training is randomly generated by Gauss distribution, setting the bias to 0. Then, the output value calculated is the forward propagation and update parameters are the back propagation. The depth of the neural network structure is in Figure 2() and is further detailed in Table 2. Because the parameters of DNN are too prone to overfitting [16], fine-tuning [17], increasing the data volume, and regularization [18] are needed to solve it.

### 3.3. Stacked Autoencoder (SAE)

**Figure 3:**
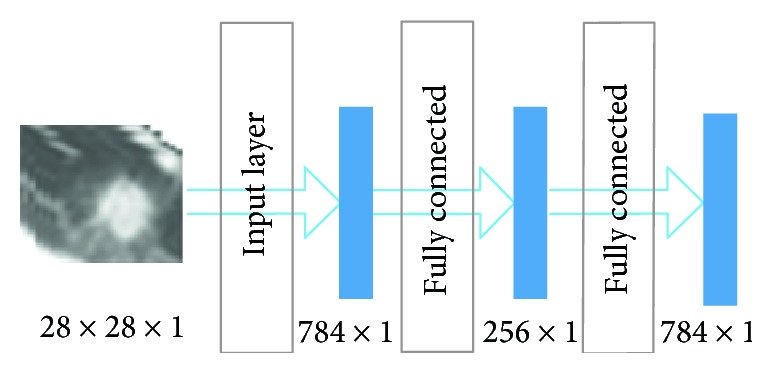
Sparse autoencoder.

A stacked autoencoder (SAE) neural network is a multilayer sparse autoencoder of a neural network. The sparse autoencoder is an unsupervised learning algorithm [19]. The sparse autoencoder is divided into three layers, namely, the input layer, hidden layer, and output layer. The number of neurons in the input and output layers is the same, and the number of hidden neurons is less than that of the input layer.  Figure 3() is the structure of the sparse autoencoder. In addition, the sparse autoencoder is divided into a coding stage and decoding stage; the coding stage is the mapping of the input layer to the hidden layer. The decoding phase is the mapping of the hidden layer to the output layer. In this paper, multiple autoencoders and softmax classifiers are combined to construct a SAE network with multiple hidden layers and a final softmax classifier [20].

**Figure 4:**
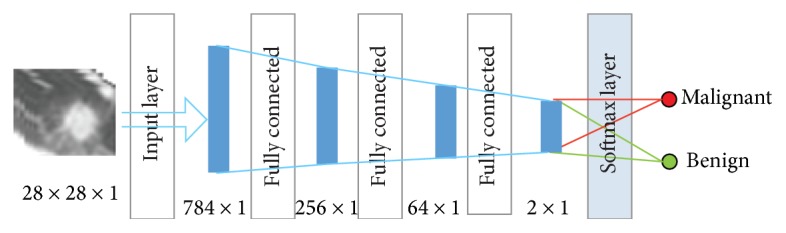
Architecture of the SAE.

Figure 4 is the structure of the stacked autoencoder neural network. The hidden layer is the hidden layer of a single sparse autoencoder. The diagnosis of lung nodules belongs to the problem of image classification; each sparse autoencoder deletes the “decode” layer after the training is completed and directly uses the encoding process for the next sparse autoencoder training of the output.

### 3.4. Loss Functions of the Neural Network

The loss function is as follows:

(1) $$\begin{array}{c} C\left(w,b\right)\equiv \frac{1}{2n}\underset{x}{\sum }{\left\|y\left(x\right)-a\right\|}^{2}+\frac{1}{2n}\lambda \underset{w}{\sum }w^{2}, \end{array}$$

where *C* is the cost function, *w* is the weight, *b* is the bias, *n* is the number of training dataset instances, *x* is the image pixel values as an input parameter, and *a* is the output value. The DNN is used to carry on the back propagation operation to modify the weight *w* and paranoid *b*, so that the difference between the predicted value and the real value is getting smaller and smaller, and thus, the accuracy is improved. The last item of the loss function is to prevent overfitting in the training process, and the sum of all weights is divided by 2*n*. Another method to prevent overfitting is dropout, which randomly shields some neurons before the back propagation, and the masked neurons do not update the parameters. Since the DNN needs a lot of data, but if a large number of data are input into the neural network, it requires a lot of memory. Therefore, in order to modify the parameters more quickly, every time a min_batch to do a back propagation.

The activation function of the neural network is Leaky ReLU, which can enhance the ability of nonlinear modeling. The ReLU activation function formula is as follows:

(2) $$\begin{array}{c} y=\left\{\begin{array}{ll} x & \text{if }x\geq 0\\ 0 & \text{if }x<0, \end{array}\right. \end{array}$$

where *x* is the result of weighted priority multiplication and paranoid addition and *y* is the output of the activation function. It can be seen that the derivative of ReLU is 0 if *x* < 0, else 1. So ReLU eliminates the problem of the gradient of the sigmoid activation function. However, with the continuous updating of the training, the weight cannot continue to be updated, which is known as “the phenomenon of neuronal death.” On the other hand, the output of ReLU is more than 0, that is, the output of the neural network is offset. The above problems can be solved using Leaky ReLU. The Leaky ReLU activation function formula is as follows:

(3) $$\begin{array}{c} y=\left\{\begin{array}{ll} x & \text{if }x\geq 0\\ ax & \text{if }x<0, \end{array}\right. \end{array}$$

where *a* is set to 0.1; *a* in Leaky ReLU is fixed and in the ReLU is not fixed.

### 3.5. LIDI-IDRI

The database used in this paper is LIDC-IDRI, which contains 244,527 images of the 1010 cases. Each subject includes images from a clinical thoracic CT scan and an associated XML file that records the results of a two-phase image annotation process performed by four experienced thoracic radiologists [13]. The distribution of thickness of CT images in lung nodules is extensive. Most of them are concentrated at 1 mm, 1.25 mm, and 2.5 mm. The size of the patient's pulmonary nodules is from 3 mm to 30 mm. The number of benign nodules with small diameter is larger, and the number of malignant nodules with larger diameter is smaller. But it is not sure that the majority of benign and malignant nodules concentrate in the 5–10 mm range.

In this paper, the location information and the degree of malignancy of pulmonary nodules in the patient's XML commentary file both can be obtained. In the XML file, four radiologists would analyze the details of the pulmonary nodules. Radiologists classify the degree of malignancy of pulmonary nodules into five categories:

1. Highly unlikely for cancer
2. Moderately unlikely for cancer
3. Indeterminate likelihood
4. Moderately suspicious for cancer
5. Highly suspicious for cancer.

The first two categories are identified as benign. The latter two categories were identified as malignant. As a total, 9106 nodular images are obtained.

### 3.6. Data Augmentation

**Figure 5:**
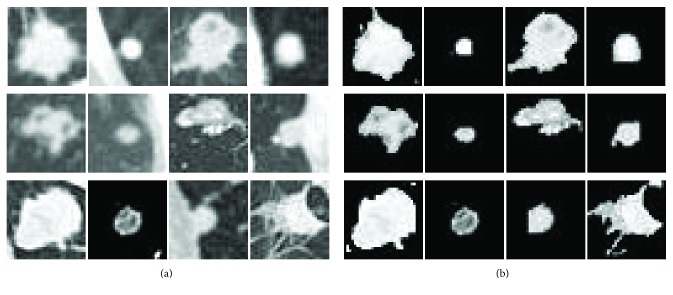
Nodular images.

It is known that the sizes of the pulmonary nodule are different. In order to obtain the textural and size characteristics of the lung nodules, the size of the pulmonary nodules is set at 28 × 28 uniformly. Firstly, the image of the pulmonary nodules was obtained by binary processing, which can obtain the approximate outline of the pulmonary nodules. Then, the value of the pulmonary nodules was restored in the proceeded image to the pixels of the pulmonary nodules. Finally, noise disturbance around pulmonary nodules can be eliminated. The original images and binary images contrast in Figure 5.

A large number of positive samples and negative samples are needed to satisfy the neural network training. In this paper, the image processing operation of translation, rotation, and flip is obtained before the image was input into the neural network, which increased the sample data of the input image. Large number of sample data can effectively improve the neural network training and testing accuracy, reduce the loss function, and ultimately improve the robustness of neural networks.

## 4. Experiments and Results

### 4.1. Experiment Setup

Caffe which is a deep learning framework made with expression, speed, and modularity in mind was used in this study. A total of 4581 images of lung nodules were used in the training. Among them, 2265 cases were benign pulmonary nodules and the other one was malignant pulmonary nodules with 2311 images. 10% of the training data set is used for cross-validation, about 448 pictures. The same data set is applied to the three different kinds of network architecture.

#### 4.1.1. Construction of the CNN

Using the network in the training stage, CNN learning rate is set to 0.01 and batch_size to 32, to get the best results. In the network, the convolution operation and the down sampling operation are carried out two times. Two convolution layers consist of 32 filters, and the kernel size is 5. The pooling layer has a kernel size of 2. The reason of using a dropout layer is to prevent overfitting. Two fully connected layers and a softmax function is following at least.

#### 4.1.2. Construction of the DNN

The DNN consists of a fully connected layer. The input image is a two-dimensional data input 28 × 28 neural network mapped into 784 × 1. The second layer is a fully connected layer of 512 × 1. The third layer is a fully connected layer of 256 × 1. After the third layer, there will be a dropout layer, with a parameter of 0.6, in which the unit will be hidden in 40%. The fourth layer is a fully connected layer of 64 × 1, whose activation function is set to ReLU.

#### 4.1.3. Construction of the SAE

The SAE is also made up of a fully connected layer. The neurons of the autoencoder's input and output are the same; the autoencoder is equivalent to the following function:

(4) $$\begin{array}{c} H_{w,b}\left(x\right)=x, \end{array}$$

where *w* and *b* are the weight and crankiness, respectively, in the neural network operation and *x* is the input parameter. The neural network is equivalent to coding the input image. Because of the problem of image classification, the hidden layer generated by the self-encoder is directly used for classification, thus canceling the decoding part of the self-encoder.

**Figure 6:**
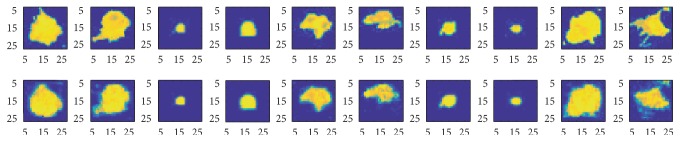
Autoencoder generates the pulmonary nodule image and original image.

During the training, the encoder-generated stack encoding is used firstly, and then, the coding part of the stack encoding network is used to apply the initializing neural network after a certain number of training to the classification. In Figure 6, the image is the contrast between the autoencoder that generates the pulmonary nodule image and original image. It is found that the image after the encoder has made the edge of the image and the characteristics of the artifacts are not obvious. So the classification accuracy will cause some loss. The detail of the SAE is in Table 3.

### 4.2. Results and Analysis

As referred in Table 4, the CNN architecture has the best precision, with an accuracy of 84.15%, sensitivity of 83.96%, and specificity of 84.32%. The accuracy of the DNN is 82.37%, the sensitivity is 80.66%, and the specificity is 83.9%. The convolution neural network obtains the good result mainly because the convolution layer operation may obtain the characteristic from the shape and the texture of two different dimensions. In different convolution kernels according to different weights for different image characteristics, a convolution kernel shared parameters in the whole process of convolution, so the convolution operation compared with fully connected operation has fewer parameters. Compared with the SAE, the DNN is not good in precision and sensitivity, but it has a better effect on specificity of 83.9%. Good specificity means that more malignant lung nodules can be detected in the same data set, which may be of a greater help in the early diagnosis of pulmonary nodules. But to a certain extent, the DNN increases the number of false-positive pulmonary nodules. The SAE and DNN are consisting only of fully connected networks, but there are different ways of generating. The SAE is generated through sparsing since the encoder training; the DNN is generated through the fully connected layer directly since training.

**Figure 7:**
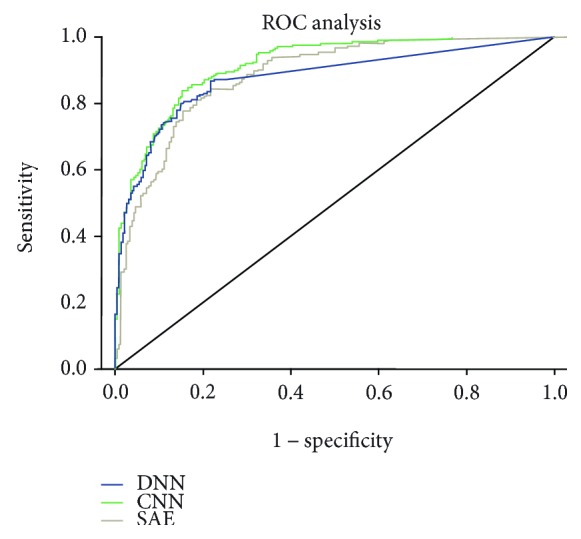
ROC curve of different neural networks.

In order to compare the performance of the neural network, the ROC curve is used in the paper.  Figure 7 is the comparison of the ROC curves of the three different neural network architectures, from which we can see that the performance of the CNN is better than that of the SAE. The AUC of the CNN is 0.916, of the SAE is 0.884, and of the DNN is 0.877.

Table 5 shows some of the relevant work and the results of this comparison. In order to increase the comparability, the experiments in the paper are done in the same data set, as well as the comparison of the same parameters. By contrast, the experimental data and the results of the CNN architecture have made some progress.

## 5. Conclusion

In this paper, three important deep neural networks were exploited and extensively evaluated. The prediction in the classification of benign and malignant pulmonary nodules was compared in LIDC-IDRI. The experimental results suggest that the CNN archived the best performance than the DNN and SAE. The layers of the neural network in this paper are relatively small, due to the limitations of the data sets. The proposed method can be expected to improve accuracy of the other database. The method can be generalized to the design of high-performance CAD systems for other medical imaging tasks in the future.

## Figures and Tables

**Table 1:** Parameter of the CNN.

Layer	Type	Input	Kernel	Output
1	Convolution	28 × 28 × 1	5 × 5	24 × 24 × 32
2	Max pooling	24 × 24 × 32	2 × 2	12 × 12 × 64
3	Convolution	12 × 12 × 64	5 × 5	8 × 8 × 64
4	Max pooling	8 × 8 × 64	2 × 2	4 × 4 × 64
5	Fully connected	4 × 4 × 64	4 × 4	512 × 1
6	Fully connected	512 × 1	1 × 1	2 × 1
7	Softmax	2 × 1	N/A	Result

**Table 2:** Parameter of the DNN.

Layer	Type	Input	Output
1	Input	28 × 28 × 1	784 × 1
2	Fully connected	784 × 1	512 × 1
3	Fully connected	512 × 1	256 × 1
4	Fully connected	256 × 1	64 × 1
5	Fully connected	64 × 1	2 × 1
6	Softmax	2 × 1	Result

**Table 3:** The structure of the SAE.

Layer	Type	Input	Output
1	Input	28 × 28 × 1	784 × 1
2	Fully connected	784 × 1	256 × 1
3	Fully connected	256 × 1	64 × 1
4	Fully connected	64 × 1	2 × 1
5	Softmax	2 × 1	Result

**Table 4:** Results for all architectures.

Models	Accuracy	Sensitivity	Specificity
CNN	84.15%	83.96%	84.32%
DNN	82.37%	80.66%	83.9%
SAE	82.59%	83.96%	81.35%

**Table 5:** Comparison with other papers.

Work	Database (samples)	Accuracy (%)	Sensitivity (%)	Specificity (%)
Nascimento et al. [21]	LIDC (73)	92.78	85.64	97.89
Orozco and Villegas [22]	NBIA-ELCAP (113)	N/A	96.15	52.17
Krewer et al. [7]	LIDC-IDRI (33)	90.91	85.71	94.74
Dandil et al. [23]	Private (128)	90.63	92.30	89.47
Parveen and Kavitha [24]	Private (3278)	N/A	91.38	89.56
Kuruvilla and Gunavathi, 2014 [6]	LIDC (110)	93.30	91.40	100
Gupta and Tiwari [25]	Private (120)	90	86.66	93.33
Hua et al. [10]	LIDC (2545)	N/A	73.30	78.70
Kumar et al. [8]	LIDC (4323)	75.01	83.35	N/A
da Silva [26]	LIDC-IDRI (8296)	82.3	79.4	83.8
CNN (this paper)	LIDC-IDRI (5024)	84.15%	83.96%	84.32%
DNN (this paper)	LIDC-IDRI (5024)	82.37%	80.66%	83.9%
SAE (this paper)	LIDC-IDRI (5024)	82.59%	83.96%	81.35%
